# The amino acid transporters SLC38A3–SLC38A5–SLC38A7 as novel anti-cancer therapeutic targets

**DOI:** 10.3389/fmolb.2026.1959729

**Published:** 2026-10-13

**Authors:** Nektarios E. Madenis, Martina Chiu, Massimiliano G. Bianchi, Chiara Ughini, Mattia E. Castellucchio, Ovidio Bussolati, Giuseppe Taurino

**Affiliations:** 1 Laboratory of General Pathology, Department of Medicine and Surgery, University of Parma, Parma, Italy; 2 MRH - Microbiome Research Hub, University of Parma, Parma, Italy

**Keywords:** amino acid transport system, asparagine, cancer, glutamine, stromal cells, tumor microenvironment

## Abstract

System N amino acid transporters SLC38A3 and SLC38A5 are carriers capable of mediating net inward or outward fluxes of substrates, such as glutamine, asparagine and histidine, coupled with Na^+^ symport and H^+^ antiport. The third member of the group, SLC38A7, principally exports glutamine and asparagine from lysosomes and its ion-coupling mechanism remains incompletely defined. Although historically less investigated than other amino acid transporters, emerging evidence reveals that System N carriers play diverse and context-dependent roles across several solid tumors and hematological malignancies. SLC38A3 can exert pro-tumor or tumor suppressive activities, displaying bidirectional transport and lineage-specific regulatory functions. SLC38A5 sustains macropinocytosis, pro-growth pathways, and antioxidant stress response conferring ferroptosis resistance. In addition, when expressed on stromal cells, it promotes amino acid exchanges within tumor microenvironment. SLC38A7 is expressed on lysosomal membranes to export proteolysis-derived amino acids and activates growth signaling. Together, evidence thus far available indicates that these transporters support metabolic plasticity, tumor progression, therapy resistance and microenvironmental remodeling in many types of cancers. This review summarizes recent advances in the comprehension of expression, regulation and functional relevance of System N transporters, highlighting their metabolic role in cancer and discussing their possible exploitation as emerging therapeutic targets.

## 1 Introduction

Amino acid transporters are critical regulators of metabolic plasticity in cancer, enabling malignant cells to sustain growth, survive during nutrient stress, and remodel their microenvironment (Hushmandi et al., 2024). Among these carriers, SLC38A5 (coding for SNAT5) (Taurino et al., 2023), SLC38A3 (coding for SNAT3) (Rubio-Aliaga and Wagner, 2016) and SLC38A7 (coding for SNAT7) (Hagglund et al., 2011) are sodium-dependent neutral amino acid transporters belonging to System N subgroup of the SLC38 family. A distinctive feature of System N transporters is their ability to mediate net inward or outward fluxes of neutral amino acids, such as glutamine, asparagine and histidine, through a coupled Na^+^ symport and H^+^ antiport. Transport direction depends on Na^+^ gradients, H^+^ coupling and amino acid availability in the intracellular and extracellular environments.

Although historically less investigated than other amino acid transporters such as SLC1A5 (ASCT2), SLC7A5 (LAT1) and SLC7A11 (xCT) (Hushmandi et al., 2024), emerging evidence highlights a significant role for System N carriers across several solid tumors and hematological malignancies, mainly for their capacity to transport glutamine and asparagine, which are two key amino acids for various types of cancers (Hanada et al., 2025; Zou et al., 2025). The activities of these carriers are highly context-dependent, varying according to oncogenic signaling, nutrient availability, and microenvironmental cues such as pH and interaction with stromal cells. Through their functional plasticity, and highly regulated expression, System N transporters support cancer metabolism, tumor progression, therapy resistance and contribute to the establishment of a pro-tumorigenic microenvironment, positioning them as novel exploitable metabolic vulnerabilities.

These aspects have been partially discussed for SLC38A5 (Sniegowski et al., 2021; Taurino et al., 2023), while remains largely unexplored for SLC38A3 and SLC38A7. This contribution summarizes recent advances on the expression, regulation and function of the three carriers across diverse cancer types. Emphasis will be placed on their roles in metabolic support to cancer cells and in the modulation of key pathways involved in proliferation and survival. We also discuss the emerging role of SLC38A5 in tumor–stroma metabolic crosstalk. Finally, we outline potentially available therapeutic strategies targeting System N transporters and evaluate them as actionable metabolic targets in oncology.

## 2 SLC38A3: a context-dependent amino acid transporter in cancer

SLC38A3 is widely expressed in physiological tissues such as liver, kidneys, brain and placenta, reflecting its central role in glutamine homeostasis, redox balance and energy metabolism. By adjusting amino acid fluxes according to nutrient availability and pH, SLC38A3 supports biosynthetic pathways, mTORC1 signaling and intercellular glutamine exchange, although its regulatory mechanisms remain poorly understood. (Rubio-Aliaga and Wagner, 2016; Gyimesi et al., 2025). In the brain, SLC38A3 is expressed in glial cells and mediates glutamine release, supplying neurons with the precursor required for glutamate synthesis and sustaining the glutamate–glutamine cycle. In the liver, SLC38A3 contributes to nitrogen metabolism and detoxification. In periportal hepatocytes, the transporter mediates glutamine uptake, which is deamidated to generate ammonia that enters the urea cycle, while SLC38A3 operates the secretion of newly synthesized glutamine from perivenous hepatocytes. In the kidney, SLC38A3 is highly upregulated during the adaptive response to metabolic acidosis, thus increasing glutamine uptake by proximal tubular cells and ammoniagenesis. Under basal conditions, SLC38A3 is predominantly localized to the basolateral membrane of the late (S3) proximal tubule, whereas during chronic metabolic acidosis the expression markedly increases at earlier (S2) tubule segments. The transporter mediates glutamine uptake from the peritubular circulation, and glutaminolysis is used to obtain ammonium and bicarbonate. Ammonium contributes to urinary acid excretion, while newly generated bicarbonate is returned to the circulation to restore systemic acid-base balance (Karinch et al., 2002; Solbu et al., 2005; Moret et al., 2007; Busque and Wagner, 2009). In the early response, extracellular acidification enhances transcription of SLC38A3 expression through the activation of a second pH-sensitive Sp1 binding site in addition to a constitutive Sp1 binding site, while during chronic adaptation SLC38A3 mRNA is stabilized through a pH-response element (Balkrishna et al., 2014). Moreover, glucocorticoids (Karinch et al., 2007) and NRF2 (Lister et al., 2018) contribute to induce SLC38A3.

The crucial physiological role of SLC38A3 is demonstrated by its loss of function mutation, which alters the amino acid homeostasis in liver, kidney and brain and cannot be compensated by other transporters, thus causing an early lethality in mice (Chan et al., 2016). Pathogenic biallelic variants of SLC38A3 in humans cause developmental and epileptic encephalopathy. Ten individuals from seven unrelated families carrying deleterious biallelic SLC38A3 variants showed global developmental delay, intellectual disability, hypotonia and absent speech, while microcephaly, epilepsy and visual impairment occurred in most of them. Metabolomic analyses identified perturbations in glutamate, histidine and nitrogen metabolism in plasma, urine and cerebrospinal fluid, supporting disrupted glutamine homeostasis as a major disease mechanism (Marafi et al., 2022). An independent family carrying the homozygous nonsense variant p.Arg454* displayed a severe phenotype characterized by very early-onset refractory seizures, profound developmental impairment and microcephaly, with one affected sibling dying from status epilepticus in early childhood, further supporting the pathogenic consequences of SLC38A3 loss of function (Alawbathani et al., 2023). Experimental endothelial selective SLC38A3 deficiency in mice reduced blood-to-brain glutamine influx and brain glutamine, thus impairing the contribution of the amino acid to glutamate and GABA metabolism and reproducing the progressive microcephaly observed in affected humans (Radzishevsky et al., 2026).

SLC38A3 tumorigenic role is ambivalent since it could exert either pro-tumor or tumor-suppressive pattern across human cancers depending on the context, which varies according to tumor type, transport directionality, oncogenic drivers and microenvironmental stressors.

In several carcinoma types, like triple-negative breast cancer (TNBC), colorectal cancer (CRC) and endometrial cancer (EC), SLC38A3 is upregulated and promotes malignant phenotypes. In TNBC, SLC38A3 promotes tumor growth and metastasis *in vivo*, while reducing apoptosis and oxidative stress. Mechanistically, *in vitro* the transporter contributes to maintain intracellular levels of different amino acids such as glutamine as well as glutathione production. Moreover, SLC38A3 inhibits GSK3β thereby increasing β-catenin signaling activation. In turn, β-catenin upregulates epithelial-mesenchymal transition (EMT) genes, thus promoting metastasis (Tan et al., 2024). In CRC, silencing of SLC38A3 in HCT116 cells reduces proliferation and migration. Based on correlation analyses, the authors proposed that SLC38A3 may positively regulate DNAJC5, thereby influencing the activity of the heat-shock chaperone Hsp70 (Zhang et al., 2024). In EC, machine learning and bioinformatic analyses identify SLC38A3 as a prognostic marker associated with tumor grade and stage (Wang et al., 2024; Jiang et al., 2025). In these studies, SLC38A3 silencing in EC cell lines reduces proliferation, migration and glycolysis *in vitro* and growth in a subcutaneous murine model, although the underlying mechanisms remain to be elucidated. Although SLC38A3 is required for normal brain development and is highly expressed in astrocytes and endothelial cells of the blood–brain barrier, where it mediates, respectively, glutamine release (Broer et al., 2004) and blood-to-brain glutamine influx (Radzishevsky et al., 2026), its functional role in brain cancer remains unclear. To date, increased mRNA expression and positive immunostaining have been reported in glioblastoma (GBM) tissue (Sidoryk et al., 2004). More recently, targeted proteomics revealed that SLC38A3 is more abundant in microvessels of human GBM than in normal brain (Bao et al., 2026), suggesting a possible adaptive response to altered amino acid requirement of proliferating GBM cells.

Conversely, SLC38A3 is downregulated in other tumors. In esophageal squamous cell carcinoma (ESCC), SLC38A3 is the most frequent copy number alterations - lost SLC38 family member in an integrated genomic cohort and is downregulated in human tumor tissue. Moreover, its suppression correlates with lymph-node metastasis, advanced stage and poor survival (Liu et al., 2020). The authors propose that SLC38A3 could act as an EMT suppressor, because its silencing *in vitro* increase levels of the mesenchymal marker SNAIL. Mechanistically, SLC38A3 physically links SETDB1, and the interaction may thus impair the SETDB1-dependent transcription of SNAIL. Consistently, the depletion of the transporter promotes proliferation, invasion and EMT of ESCC cell lines *in vitro* (Liu et al., 2020). SLC38A3 has also been reported as downregulated in gastric cancer *in silico* analysis, although functional activity has not been investigated (Hannan et al., 2024).

SLC38A3 also displays context-dependent and model-dependent role heterogeneity, particularly across lung cancer models with different cellular backgrounds, genetic alterations and environmental stressors. In Non-Small Cell Lung Cancer (NSCLC), SLC38A3 is upregulated in human tumor tissue, increases from primary to metastatic lesions, and its expression is associated with poorer prognosis (Wang et al., 2017). A The Cancer Genome Atlas (TCGA)-based analysis also identified SLC38A3 among prognosis-associated genes in lung adenocarcinoma (LUAD), the most prevalent subtype of NSCLC (Xu et al., 2020). Mechanistically, in A549 cells SLC38A3 reduces intracellular glutamine and histidine, consistent with its efflux activity, thus activating 3-phosphoinositide-dependent protein kinase-1 (PDK1) and the downstream AKT, and increasing levels of the EMT marker SNAIL. AKT inhibition reverses SLC38A3-induced EMT-marker changes, supporting the hypothesis that SLC38A3 activate AKT/GSK3β/β-catenin pathway. Consistently, SLC38A3 overexpression promotes lung metastasis *in vivo* and is associated with increased AKT phosphorylation (Wang et al., 2017). Notably, depletion of glutamine and histidine similarly activates this pathway. Although AKT activation in response to amino-acid deprivation has been previously described in NSCLC (Jin et al., 2021), SLC38A3-dependent activation of PDK1 has been reported for the first time in (Wang et al., 2017). In contrast, multi-omics analysis of TCGA data proposes SLC38A3 as a tumor-suppressive factor in LUAD, associating its expression with improved prognosis. Consistently, SLC38A3 silencing in NCI-H1395 cells reduces the percentage of apoptotic cells compared with control conditions, although the underlying mechanism remains undefined (Zhou et al., 2025). Moreover, chronic cadmium exposure induces cancer-like features in lung epithelial cell lines and lowers the expression of SLC38A3 protein, indicating that transporter downregulation accompanies this transformation like phenotype (Person et al., 2013). However, the evidence supporting a pro-tumor role in lung cancer includes functional transport analyses, experiments in multiple cell lines and *in vivo* metastatic models, whereas the proposed tumor-suppressive role in LUAD currently relies mainly on multi-omics associations and functional evaluation in a single cell model. The attribution of a tumor suppressive role for SLC38A3 should, therefore, be considered preliminary and strictly model-dependent.

In hepatocellular carcinoma (HCC), SLC38A3 involvement also appears context-dependent. In a study, SLC38A3 mRNA is less expressed in murine HCC tumors than normal murine liver and in human HCC cell lines than in primary hepatocytes, and its lower expression correlates with worse patient survival (Tambay et al., 2024). In contrast, genome-wide screening shows that a single nucleotide polymorphism in an HNF4α-binding site of the DEAF1 promoter disrupts HNF4α binding, leading to DEAF1 overexpression. In turn, DEAF1 induces SLC38A3, which promotes HCC progression. (Huang L. et al., 2025). The mechanism was validated *in vitro* in DEAF1-silenced HepG2 and Huh7 cells: DEAF1 is needed to induce SLC38A3, which reduces ROS levels, supporting glutathione synthesis likely through glutamine uptake, although the functional activity of the transporter was not investigated (Huang L. et al., 2025).

Together, these findings indicate that SLC38A3 expression alone is not sufficient to predict its pro-tumor or tumor-suppressive role. Rather, its pro-tumor functional impact emerges from the specific metabolic, transcriptional and lineage-dependent programs active within each cancer type.

## 3 SLC38A5: a multifunctional metabolic player in cancer and stromal cells

SLC38A5 is expressed in periportal hepatocytes and pancreatic acinar cells, where it mediates glutamine uptake, contributing to nitrogen handling and metabolic homeostasis. In the brain, SLC38A5 is expressed in glial cells and, together with SLC38A3, supports glutamine secretion to sustain the glutamate–glutamine cycle between glial cells and neurons (Taurino et al., 2023; Gyimesi et al., 2025). Notably, SLC38A5 is transcriptionally regulated by Myc (Wise et al., 2008), inflammatory stimuli (Alasoo et al., 2015) and hypoxia (Taurino et al., 2022), which collectively can explain why it is overexpressed across multiple human cancers. Beyond glutamine and asparagine, SLC38A5 can transport other amino acids, such as serine and glycine, further expanding its potential metabolic relevance. Indeed, all these amino acids support anaplerosis, lipid biosynthesis and the one-carbon cycle required for nucleotide synthesis (Sniegowski et al., 2021; Taurino et al., 2023). Through its Na^+^/H^+^ exchange activity, SLC38A5 exports protons during amino acid uptake, contributing to intracellular alkalinization and extracellular acidification. Alkalinization promotes cytoskeletal remodeling and macropinocytosis, enabling cancer cells to scavenge extracellular proteins as an additional nutrient source (Bhutia et al., 2022). Recent evidence in colon, pancreatic and bone cancers indicates that SLC38A5-mediated amino acid uptake activates mTORC1 and confers resistance to ferroptosis by sustaining glutathione synthesis (Kim et al., 2023; Sniegowski et al., 2023; Huang et al., 2024; Zhang et al., 2026).

SLC38A5 is overexpressed across all the major subtypes of breast cancer (BC), with the highest levels in TNBC patient-derived xenograft cell lines, and a mouse model of spontaneous mammary tumor (Ramachandran et al., 2021). Its expression correlates with poor survival, reduced cisplatin sensitivity and increased proliferation both *in vitro* and *in vivo* (Mathew et al., 2024; Shen et al., 2024). Mechanistically, SLC38A5 mediates the uptake of glutamine, serine and glycine, sustaining metabolism and activating macropinocytosis (Ramachandran et al., 2021; Shen et al., 2024). It can also mediate the uptake of the selenium-containing methionine, which activates NRF2 and induces the expression of SLC7A11. Selenium is also required for the activity of GPX4, the key enzyme for the prevention of lipid peroxidation. These effects allow TNBC cells to counteract ferroptosis (Mathew et al., 2024).

In pancreatic ductal adenocarcinoma (PDAC) patients, SLC38A5 expression correlates with higher tumor grade, poor prognosis and gemcitabine resistance (Kim et al., 2023; Sennoune et al., 2023; Sniegowski et al., 2023). Functionally, the transporter can mediate the uptake of glutamine, serine, glycine and, possibly, alanine, supporting macropinocytosis (Sennoune et al., 2023), mTOR signaling (Sniegowski et al., 2023) and glutathione synthesis (Kim et al., 2023), thus promoting cancer cell growth and ferroptosis resistance *in vitro* and in mice. Although no direct regulatory link between KRAS and SLC38A5 has been demonstrated, at variance with CRC (see below), KRAS-mutant PDAC displays metabolic traits (glutamine dependence, macropinocytic nutrient acquisition, mTOR activation and redox stress) that make SLC38A5 potentially important for KRAS-driven programs.

In CRC, SLC38A5 is tightly regulated and promotes proliferation, migration, invasion, ferroptosis resistance and chemoresistance to oxaliplatin, 5-fluorouracyl and irinotecan (Nguyen et al., 2024; Bartolome et al., 2025; Huang Y. et al., 2025; Zhang et al., 2026). In CRC cell lines, SLC38A5 expression is induced by the transcription factor ETV4 (Huang Y. et al., 2025), KRAS activation (Nguyen et al., 2024), and CDH17/Wnt/β-catenin signaling, which activates Myc that, in turn, upregulates SLC38A5 (Bartolome et al., 2025). Mechanistically, the transporter promotes macropinocytosis (Nguyen et al., 2024; Bartolome et al., 2025) and enhances resistance to ferroptosis by mediating glutamine uptake and glutathione synthesis (Zhang et al., 2026). Moreover, SLC38A5 facilitates the nuclear translocation of YAP, a co-activator of NRF2, inducing the expression of antioxidant genes such as SLC7A11 and GPX4 (Zhang et al., 2026), although it remains unclear if this effect is associated with amino acid transport activity or transport-independent regulatory functions.

In osteosarcoma (OS), SLC38A5 is associated with tumor-grade and poor prognosis in patients, favoring cancer cell proliferation, migration and invasion (Zhang et al., 2022; Huang et al., 2024). Functionally, SLC38A5 inhibits ferroptosis and supports cancer growth of OS cell lines and subcutaneous xenograft murine models through the uptake of glutamine. Mechanistically, glutamine fuels glutathione synthesis and activates P13K/AKT/mTOR pathway, which enhances the SREBP1/SCD-1 signaling, thus preventing reactive oxygen species accumulation (Huang et al., 2024). Moreover, glutamine sustains OXPHOS metabolism and preserves NADPH/NADP^+^ balance, enabling anoikis resistance of OS and chondrosarcoma cells. In both cancer types, treatment with melatonin suppresses SLC38A5 expression, disrupts glutamine metabolism, inhibits PI3K–AKT signaling, increases oxidative stress, and restores anoikis sensitivity, reducing lung metastasis *in vivo* (Nguyen et al., 2026).

Additional evidence suggests roles of SLC38A5 in ovarian cancer, where the transporter has been identified *in silico* as a potential prognostic parameter (Zhang et al., 2023), and in oral squamous cell carcinoma (OSCC), where neoplastic cells internalize M2-macrophage–derived glutamine through SLC38A5, thus enhancing proliferation and invasion by activating the FOXM1/CNIH4 axis (Liu et al., 2026). Evidence in other Myc-associated tumors such as HCC and prostate cancer (PCa) is still limited. In HCC, SLC38A5 is weakly detectable *in vitro* and in tumor tissue, except for HepG2 cells, and its expression is not associated with survival outcomes (Tambay et al., 2024). In androgen receptor-positive PCa LNCaP cells, SLC38A5 is upregulated by dihydrotestosterone (Okudaira et al., 2014), but its functional role in these cells, as well as in androgen-insensitive PCa cells, remains unknown.

SLC38A5 plays a key pro-tumor role also in hematological malignancies. It is exploited together with SLC1A5 by asparagine-auxotrophic B-cell precursor ALL (BCP-ALL) cells to internalize the amino acid, thereby activating mTOR signaling and supporting growth (Taurino et al., 2024). SLC38A5 is detectable also in malignant plasma cells from multiple myeloma (MM) patients, with expression increasing from MGUS to MM and plasma cell leukemia (Bolzoni et al., 2016). Finally, the transporter is upregulated by the RNA helicase DHX15 in T-cell acute lymphoblastic leukemia (T-ALL) lymphoblasts (Guo et al., 2023), although its functional role in this malignancy remains uninvestigated.

Importantly, SLC38A5 is also expressed in the microenvironment of several types of tumors, where it can regulate amino acid availability. Within the bone marrow niche, B-ALL blasts establish a pro-tumor amino acid exchange with primary bone marrow mesenchymal stromal cells (MSCs): leukemic blasts secrete glutamine, taken up by MSCs and converted into asparagine, which is then exported through SLC38A5 and provided to ALL cells. This mechanism protects leukemic blasts against asparagine depletion during the treatment with L-asparaginase, one of the mainstays of B-ALL chemotherapy, and is prevented if SLC38A5 is silenced or pharmacologically inhibited (Chiu et al., 2021). A similar mechanism occurs in glutamine-addicted MM cells: they release glutamate via SLC7A11, MSCs internalize glutamate through SLC1A1 and convert it into glutamine, subsequently secreted through SLC38A5 to sustain neoplastic cell growth (Taurino et al., 2026b). Notably, in both B-ALL and MM, SLC38A5 is upregulated in MSCs by co-culture with cancer cells and, in both cases, it exerts pro-tumor functions mediating amino acid efflux.

SLC38A5 role is less characterized in the microenvironment of solid tumors. In clear cell renal cell carcinoma (ccRCC), the transporter is more expressed in cancer-associated fibroblasts (CAFs) than in normal fibroblasts, correlates with poor prognosis, and sustains the pro-tumoral activity of CAFs. Consistently, SLC38A5 silencing in CAFs suppresses proliferation, migration, and invasion of ccRCC cell lines cultured in CAFs-conditioned medium. Transport activity has not been assessed; however SLC38A5-silencing lowers the secretion of CCL5, highlighting a stromal SLC38A5–CCL5 axis as a potential therapeutic target (Chen et al., 2023). Under conditions mimicking hepatic fibrosis, a potentially pro-tumorigenic state, activated human hepatic stellate LX-2 cells display Myc-dependent SLC38A5 induction (Wu et al., 2024).

As far as non-stromal cells are concerned, SLC38A5 localizes to lysosomes of dendritic cells, where it potentiates psoriatic inflammation by mediating H^+^ influx with glutamine and asparagine efflux. This promotes lysosomal acidification, with consequent TLR7 activation and NF-κB–dependent induction of IL-23 and IL-1β (Zhu et al., 2023). SLC38A5 expression has also been reported in human macrophages activated by LPS (Alasoo et al., 2015; Cobo et al., 2022; Taurino et al., 2026a). In particular, M1-polarized macrophages stimulated with LPS/IFNγ upregulate SLC38A5 at later times of treatment. The change is associated with increased glutamine synthesis and secretion (Taurino et al., 2026a). These findings indicate that macrophages can potentially modify extracellular amino-acid availability through SLC38A5, thereby influencing the behavior of other cells within the tumor microenvironment. Notably, SLC38A5 is the only SLC38 family member upregulated in M2 macrophages, which supply glutamine to OSCC cells; however, the demonstration of glutamine secretion through SLC38A5 has not yet been obtained in that model (Liu et al., 2026). Endothelial cells also express SLC38A5, which mediates serine and glutamine uptake across the blood–brain barrier (BBB), supporting brain development in mice (Hamada et al., 2023; Radzishevsky et al., 2023). L-serine is critical for neurodevelopment and thought to be synthesized exclusively from glucose. Recently, SLC38A5 has been identified as the main transporter of L-serine across the BBB during early postnatal life of mice. Consistently, SLC38A5 knockout decreases brain levels of L-serine and, consequently, of neuronal racemase-derived D-serine, without altering the levels of other amino acids. This leads to metabolic, synaptic and mitochondrial abnormalities accompanied by motor impairments (Radzishevsky et al., 2023). In humans, transcriptomic profiling of endothelial colony-forming cells from patients with proliferative retinopathy identified SLC38A5 among the upregulated angiogenesis-related genes (da Silva Costa et al., 2024). Norrin factor binds the Frizzled-4 receptor and, in the presence of the co-receptor LRP5, activates the canonical Wnt/β-catenin pathway. In turn, β-catenin induces the expression of SLC38A5 that favors the uptake of glutamine, which supports the expression of VEGFR2 on the cell membrane (Wang et al., 2022). These data suggest a role for SLC38A5 in pathological neovascularization and as a potential anti-angiogenic target.

In summary, the dual expression in malignant and non-malignant compartments positions SLC38A5 as a multifunctional metabolic hub within the tumor ecosystem.

## 4 SLC38A7: a lysosomal transporter supporting cancer metabolism, signaling and progression

SLC38A7 was initially identified as a neuronal glutamine transporter and proposed to mediate the uptake of glial-derived glutamine, thereby supporting neuronal synthesis of glutamate (Hagglund et al., 2011; Gyimesi et al., 2025). However, in cancer cells, at variance with SLC38A3 and SLC38A5, SLC38A7 is predominantly localized on lysosomal membranes, where it mediates the efflux of proteolysis-derived glutamine and asparagine into the cytosol. Through this activity, SLC38A7 acts as a metabolic gatekeeper linking lysosomal nutrient recycling, derived mainly from extracellular proteins, to mTORC1 activation, supporting cell growth under nutrient limitation (Verdon et al., 2017). The most direct pro-tumor functional data derive from studies on pancreatic cancer, while evidence in gastric cancer suggest additional regulatory and signaling functions.

Under glutamine restriction, pancreatic cancer MIA PaCa-2 cells maintain proliferation by internalizing albumin via macropinocytosis; albumin is degraded in lysosomes, and the resulting amino acids are exported to the cytosol through SLC38A7 (Verdon et al., 2017). In KRAS-driven MIA PaCa-2 cells, this pathway activates mTORC1 through a Rag-independent mechanism, reinforcing the role of SLC38A7 as a lysosomal gatekeeper that couples nutrient salvage to growth signaling (Meng et al., 2022). Consistently, SLC38A7 expression is higher in PDAC than in normal pancreatic tissue (Meng et al., 2022). Notably, SLC38A7 was identified as the principal lysosomal glutamine and asparagine exporter also in HeLa cells (Verdon et al., 2017). In addition, *in silico* analyses indicate SLC38A7 as a prognostic gene in cervical cancer (Luo et al., 2026).

In gastric cancer SLC38A7 is associated with poor prognosis, as indicated in the TCGA transcriptomic datasets and tumor-tissue analyses, and promotes proliferation, migration, invasion, mitochondrial function and tumor growth in both *in vitro* and *in vivo* experimental models (Yu et al., 2022; Hua et al., 2024; Li K. et al., 2026). SLC38A7 expression and its pro-tumor effects are promoted by METTL3-dependent N6-methyladenosine modification and IGF2BP2-mediated stabilization of its transcript (Hua et al., 2024). Moreover, SLC38A7 interacts with myosin heavy chain 9 (MYH9), facilitating MYH9-dependent GSK3β ubiquitination and degradation and thereby activating Wnt/β-catenin signaling (Li K. et al., 2026).

In ER-positive breast cancer, machine-learning and bioinformatic analyses identify SLC38A7 within prognostic metabolic gene sets, without SLC38A7-specific functional validation (Chen et al., 2025; Yuan et al., 2026). In addition, separate studies implicate SLC38A7 in tumor-cell adaptation to anthracyclines and in the pharmacogenetics of anastrozole exposure. SLC38A7 is upregulated during anthracycline-resistance evolution and is associated with poor prognosis. Its knockdown reduces cancer cell proliferation and increases apoptosis, although these effects are not resistance-specific and its transport activity was not directly assessed (Audet-Delage et al., 2023). A genome-wide association study (GWAS) and overexpression assay support SLC38A7 as a candidate determinant of anastrozole uptake and circulating drug levels, although clinical benefit was not evaluated (Dudenkov et al., 2019).

In squamous NSCLC, high SLC38A7 expression correlates with poor survival (Haratake et al., 2021). In LUAD patients, elevated expression of SLC38A7 is associated with advanced disease features and worse outcomes and is an independent prognostic factor for recurrence-free survival. Functional relevance for SLC38A7 has also been supported *in vitro*: knockdown of the transporter reduces proliferation and cause G1 arrest in A549 and H23 LUAD cells, although the underlying metabolic or signaling mechanisms remain undefined (Hashinokuchi et al., 2025). Notably, a bioinformatic analysis links SLC38A7 to methionine transport (Chang et al., 2023).

In head and neck squamous carcinoma (HNSCC), SLC38A7 is upregulated, but *in vitro* oncogenic validation are limited (Li et al., 2022). In HCC and OS, evidence is restricted to multigene prognostic analyses and does not establish a consistent independent pro-tumor role (Ying et al., 2021; Zheng et al., 2022). Together, SLC38A7 cannot yet be considered a universal cancer driver, but appears to support tumor progression in selected contexts. The implicated mechanisms vary by cancer type and include lysosomal amino acid recycling, protein-interaction-dependent signaling and adaptation to treatment-associated metabolic stress.

## 5 Therapeutic targeting of system N transporters: opportunities and challenges

Among System N transporters, SLC38A5 currently represents the most actionable therapeutic target. GluγHA (L-glutamic acid γ-monohydroxamate) is the only known direct competitive inhibitor of SLC38A5-mediated glutamine and asparagine influx and efflux (Hashim et al., 2018; Taurino et al., 2023; Taurino et al., 2024), although endowed with low affinity (IC_50_ ≈ 0.6 mM). Indirect inhibitors include amiloride, the antihelminthic drug niclosamide, which interfere with the Na^+^/H^+^ exchanger NHE-1 (SLC9A1) (Ramachandran et al., 2021; Sennoune et al., 2023), and melatonin, which downregulates SLC38A5 expression (Nguyen et al., 2026). Notably, in triple-negative breast cancer and colorectal cancer SLC38A5 upregulation is associated with SLC7A11 overexpression (see above) and, as such, would counteract ferroptosis. However, a high SLC7A11 expression may render cells vulnerable to disulfidptosis, another regulated cell death pathway recently described in other cancer models (Liu et al., 2023; Li Y. et al., 2026; Tolbatov and Marrone, 2026). Under glucose starvation, reduced availability of NADPH generated by the pentose phosphate pathway prevents the reduction of SLC7A11-imported cystine, leading to the accumulation of the amino acid. This, in turn, triggers disulfide stress through aberrant disulfide bond formation in cytoskeleton proteins, ultimately causing F-actin collapse and cell death (Liu et al., 2023). Importantly, SLC38A5 also operates within the tumor microenvironment, mediating amino-acid exchange between malignant cells and stromal compartments. This dual cancer–stroma activity suggests that SLC38A5 inhibition may simultaneously damage tumor-intrinsic metabolism and disarticulate microenvironmental nutrient support, pointing to the transporter activity as a tractable metabolic vulnerability.

In contrast, SLC38A3 and SLC38A7 remain pharmacologically undrugged. For SLC38A3, its context-dependent oncogenic versus tumor-suppressive roles complicates its therapeutic inhibition. Direct targeting would require functional biomarkers capable of identifying tumors dependent on SLC38A3 transport, while excluding contexts in which its expression may retain tumor-suppressive activity. Moreover, the essential physiological roles of SLC38A3 in the brain, kidney and the liver, together with the severe consequences of genetic loss of function (Chan et al., 2016), indicate that systemic inhibition may carry significant on-target toxicity. Although genetic deficiency does not directly predict the effects of partial or transient inhibition in adults, these findings establish a clear requirement for tumor-selective delivery, coupled with careful pharmacodynamic monitoring to ensure both efficacy and safety. For example, an interesting possibility may be provided by an Enhanced Permeability and Retention (EPR)-approach based on nanodrugs. Moreover, therapeutic pathway-level modulation would rely on indirect strategies, including interference with SLC38A3-associated pathways such as AKT or DNAJC5/Hsp70. A database-guided docking analysis identified periodate-oxidized adenosine as a putative computational hit for SLC38A3 (Wang et al., 2024), but this remains an *in silico* lead, while biochemical binding, transport-inhibition, cellular target-engagement or selectivity data are currently not available. SLC38A7 similarly lacks direct inhibitors, and its pharmacological targeting remains conceptual. Its role in lysosomal nutrient salvage provides a rationale for developing inhibitors of lysosomal glutamine and asparagine efflux or of downstream glutamine utilization. Potential indirect strategies include targeting the METTL3–IGF2BP2 regulatory axis, the SLC38A7–MYH9 interaction, downstream Wnt/β-catenin signaling or, as recently, proposed glutaminase activity (Hua et al., 2024). However, these approaches represent pathway-level intervention rather than selective inhibition of SLC38A7. Thus, they may reproduce selected consequences of SLC38A7 suppression but cannot establish the transporter itself as pharmacologically druggable. Moreover, compensatory increase of glycolysis may limit the efficacy of lysosome- and glutamine-targeted combinations (Lengauer et al., 2023).

Overall, whereas SLC38A3 and SLC38A7 remain potential metabolic vulnerabilities but lack selective pharmacological tools, SLC38A5 currently offers the most concrete therapeutic opportunities. However, even for SLC38A5, the development and characterization of high-affinity, small-molecule inhibitors is required to substantially increase the feasibility of preclinical testing in view of clinical applications (Figure 1).

**FIGURE 1:**
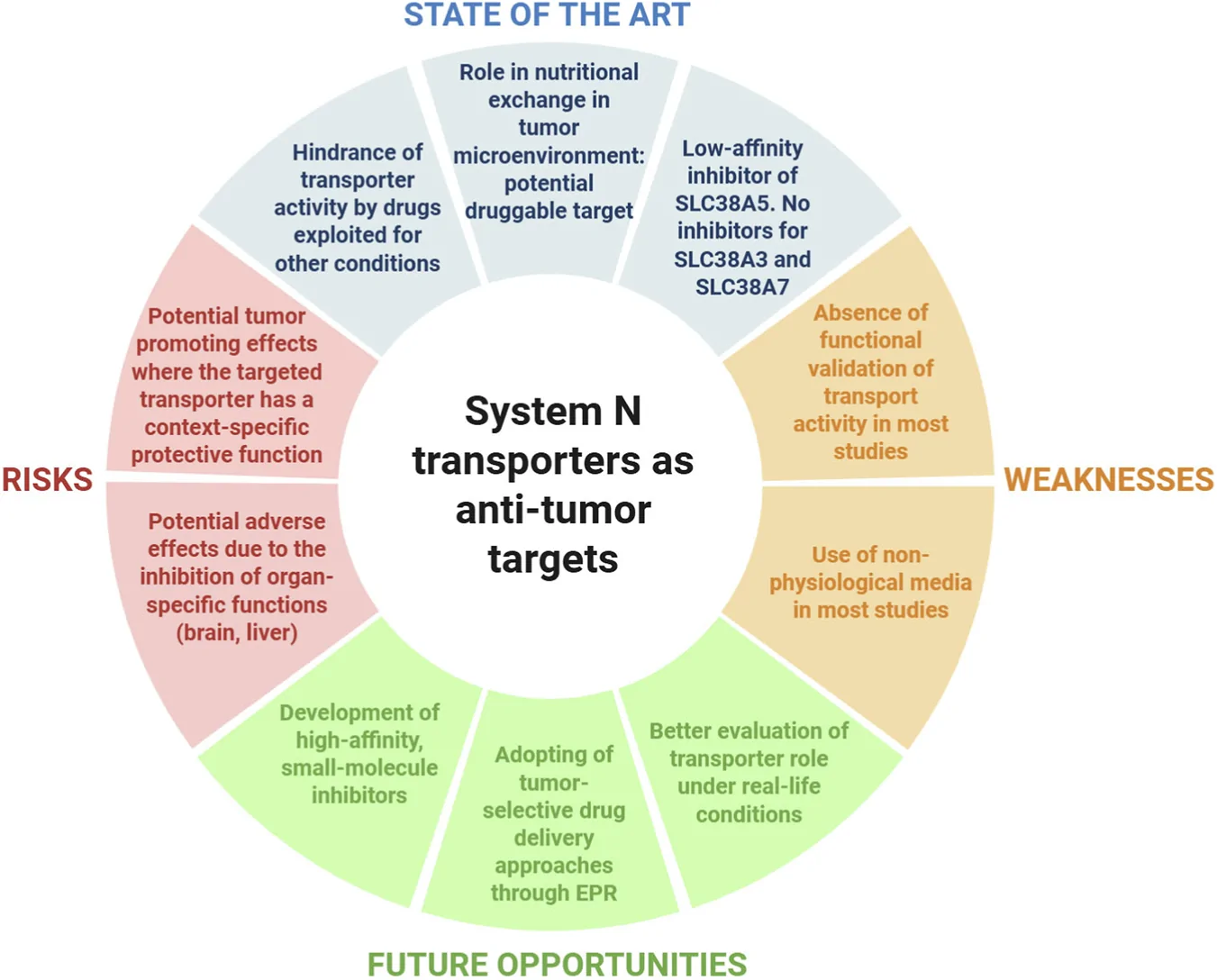
A summary diagram illustrating the state of the art, risks, weaknesses, and future opportunities related to the potential role of System N transporters as anti-tumor therapeutic targets. The figure was created with Biorender.com (Agreement number: DI2A938U2F).

## 6 Conclusions and future perspectives

System N transporters integrate shared metabolic and context-specific functions that sustain physiological activity in specific organs and the growth of several types of cancer, with the notable exception of SLC38A3, which has been reported to exert either pro-tumor or tumor-suppressive activity (Figure 2). Collectively, SLC38A3, SLC38A5 and SLC38A7 contribute to glutamine and asparagine handling, redox balance and metabolic resilience in various types of human cancers (Table 1).

**FIGURE 2:**
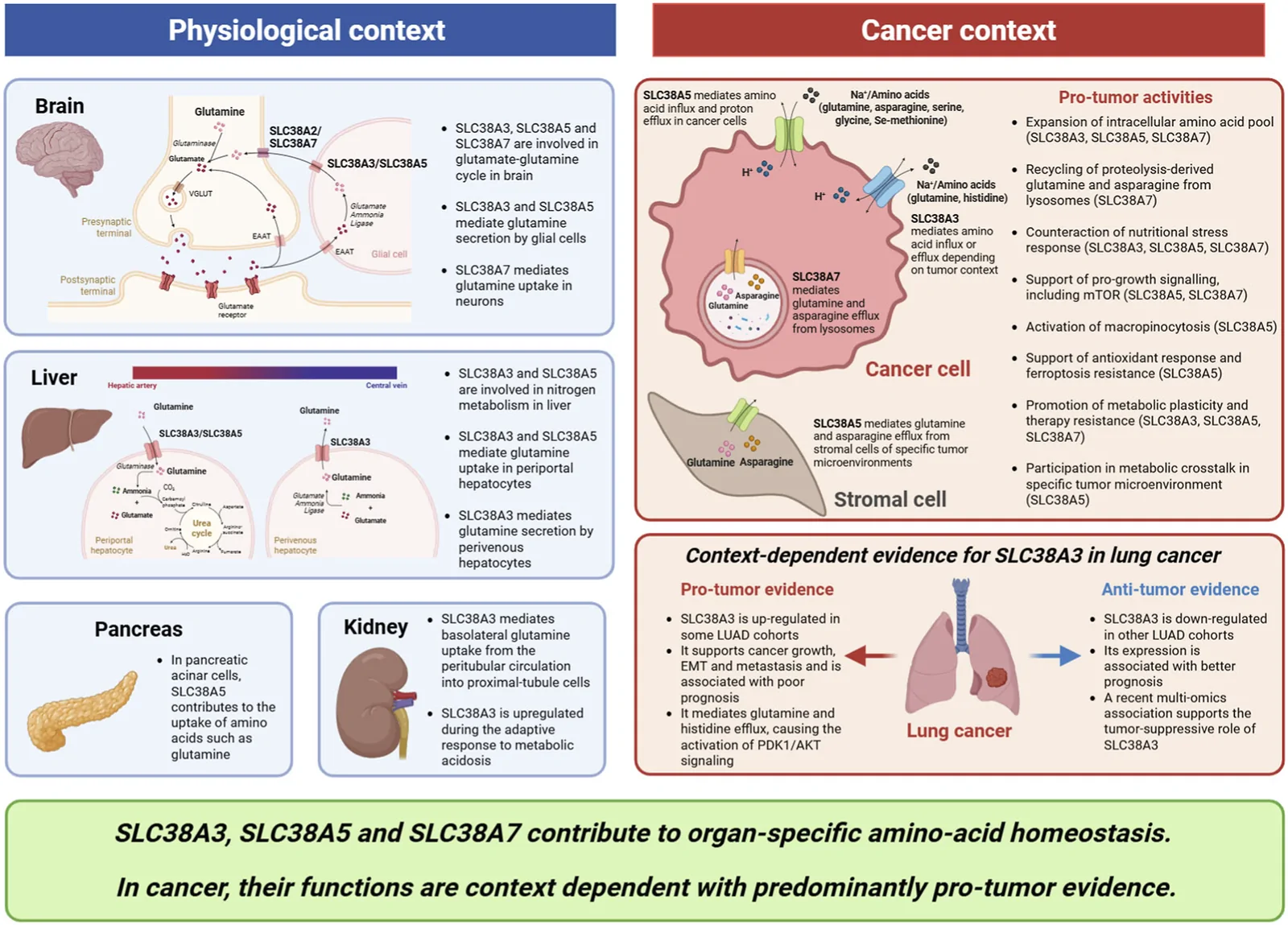
Overview of the physiological functions and cancer-associated activities of System N transporters SLC38A3, SLC38A5 and SLC38A7. The diagram summarizes their organ-specific roles in amino-acid homeostasis and highlights how their dysregulation in tumors supports metabolic adaptation, growth and therapy resistance. A dedicated panel illustrates the context-dependent role of SLC38A3 in lung adenocarcinoma. The figure was created with Biorender.com (Agreement number: HM2A938XWS).

**TABLE 1:** Summary of the pro-tumor activities mediated by System N transporters SLC38A3, SLC38A5 and SLC38A7 across different cancer types.

Transporter	Possible activities	Possible pro-cancer effects	Detection in cancer models
SLC38A3 (SNAT3)	Influx of glutamine	Glutathione production and ROS reduction	Yes: HCC (DEAF1 associated SNP) (Huang et al., 2025a)
	Maintaining intracellular amino acid levels	Glutathione production; Inhibition of GSK3β with activation of β-catenin signaling	Yes: TNBC (Tan et al., 2024)
	Efflux of glutamine and histidine	Activation of PDK1/AKT/β-catenin signaling	Yes: NSCLC (Wang et al., 2017)
	Possible positive regulation of DNAJC5	Hsp70-associated proliferation and migration	Yes: CRC (Zhang et al., 2024)
SLC38A5 (SNAT5)	Cancer cells		
	Uptake of glutamine	Fuelling of glutamine dependent pathways; glutathione synthesis for ferroptosis resistance; mTOR activation	Yes: TNBC (Ramachandran et al., 2021; Shen et al., 2024), PDAC (Kim et al., 2023; Sniegowski et al., 2023), CRC (Zhang et al., 2026), OS (Huang et al., 2024), OSCC (Liu et al., 2026)
	Uptake of asparagine	Avoidance of nutritional stress in amino acid auxotrophic cancers	Yes: B-ALL (Taurino et al., 2024)
	Uptake of serine and glycine	Biosynthesis of purine and pyrimidines	Yes: TNBC (Ramachandran et al., 2021), PDAC (Sennoune et al., 2023)
	Uptake of Se-Methionine	Activation of antioxidant factors NRF2/GPX4	Yes: TNBC (Mathew et al., 2024)
	Secretion of protons	Intracellular alkalinization with macropinocytosis activation	Yes: TNBC (Ramachandran et al., 2021), PDAC (Sennoune et al., 2023), CRC (Nguyen et al., 2024; Bartolome et al., 2025)
	Nuclear translocation of YAP	Activation of antioxidant factor NRF2	Yes: CRC (Zhang et al., 2026)
	Cells of the tumor microenvironment		
	Secretion of glutamine and asparagine by MSCs	Nutritional support of auxotrophic cancer cell growth	Yes: B-ALL (Chiu et al., 2021), MM (Taurino et al., 2026b)
	Lysosomal H^+^ influx in dendritic cells	TLR7 activation with production of pro-inflammatory cytokines	No
	Secretion of glutamine by macrophages	Modulation of amino-acid availability in the tumor microenvironment	No
	Uptake of glutamine and serine in endothelial cells	Angiogenesis	No
SLC38A7 (SNAT7)	Lysosomal glutamine and asparagine efflux	Amino acid recycling; mTOR activation	Yes: PDAC (Verdon et al., 2017; Meng et al., 2022)
	Interaction with MYH9	Activation of Wnt/β-catenin signaling	Yes: GC (Li et al., 2026a)
	Undefined mechanisms	Chemoresistance - associated proliferation	Yes: ER + BC (Dudenkov et al., 2019; Audet-Delage et al., 2023)

B-ALL: pre-B, acute lymphoblastic leukemia; CRC: colorectal cancer; GC: gastric cancer; ER + BC: estrogen-positive breast cancer; HCC: hepatocellular carcinoma; MM: multiple myeloma; NSCLC: Non-Small Cell Lung Cancer; OS: osteosarcoma; OSCC: oral squamous cell carcinoma; PDAC, pancreatic ductal adenocarcinoma; T-ALL, pre-T acute lymphoblastic leukemia; TNBC, triple-negative breast cancer.

The best-characterized functions of these carriers include plasma-membrane transport (SLC38A3, SLC38A5), macropinocytosis (SLC38A5) and nutrient recovery from lysosomes (SLC38A7). These mechanisms can operate in parallel within the same tumor, such as pancreatic cancer where SLC38A5-driven macropinocytosis combines with SLC38A7-mediated proteolysis-derived amino acid export into the cytosol, or act independently, as seen in B-ALL (SLC38A5), gastric cancer (SLC38A7) or NSCLC (SLC38A3). Importantly, System N transporter activity can be dynamically shaped by nutrient availability, transmembrane pH gradient, stromal interactions, indicating that the functional relevance of these carriers is specific for the metabolic context rather than a simple consequence of their expression level. This highlights the need for experimental approaches that better mimic physiological or disease-related conditions to assess the transport activity and regulatory potential of these carriers. In particular, standard cell-culture media differ markedly from human plasma, containing supraphysiological concentrations of several metabolites while lacking others that are present *in vivo*. Such discrepancies can profoundly bias the evaluation of metabolic dependencies, including amino-acid transport and utilization (Tardito and MacKay, 2023). In particular, the exchange rate between extracellular and intracellular environments is strongly influenced by medium composition (Taurino et al., 2022). For example, supraphysiological concentrations of certain metabolites in conventional media, such as glutamine, may artificially increase their consumption and cell dependence compared with physiological conditions. On the contrary, several conventional media lack glutamate and asparagine, thus forcing their secretion in standard media. Consistently, these variations can affect the development of nutritional stress and the resulting cellular responses.

Beyond metabolism, System N transporters also exert regulatory functions that expand their impact on tumor biology. SLC38A3 may modulate transcriptional programs through SETDB1–SNAIL-associated mechanisms (Liu et al., 2020); SLC38A7 influences β-catenin signaling via MYH9 (Li K. et al., 2026); and SLC38A5 affects antioxidant and ferroptosis pathways through YAP-NRF2 axis (Zhang et al., 2026). These emerging non-canonical roles, which are not directly linked to transport activity, suggest that System N transporters act not only as nutrient carriers but also as integrated metabolic-signaling nodes.

Among the three, SLC38A5 stands out for its microenvironmental reach and finely tuned regulatory mechanisms. Its expression in stromal, immune and endothelial cells enables amino acid exchange between malignant and non-malignant populations, supporting metabolic cooperation, and resistance to nutrient stress. This intercellular role makes SLC38A5 a central metabolic hub of the tumor microenvironment, differently from SLC38A3 and SLC38A7, whose functions remain largely confined to cancer cells.

Future progress will be required to define how System N transporters integrate metabolic fluxes, macropinocytosis, ferroptosis sensitivity and signaling networks across tumor types. To date, most studies on SLC38A3 and SLC38A7 rely on *in silico* analyses or *in vitro* evaluation of mRNA and protein levels, with limited or absent functional validation of transport activity under real-life conditions. In the case of SLC38A5, the number of studies has increased substantially in recent years and now includes more functional evidence, partly facilitated by the availability of inhibitors. Functional assays capable of measuring amino acid flux, transport directionality and metabolic state will be essential to determine the effective function of each transporter (Figure 1). Moreover, given their context-dependent roles, biomarker-guided stratification will be necessary to identify tumors in which transporters represent dominant metabolic drivers, cooperative nodes or dispensable components. Such integrated approaches will be pivotal to uncover metabolic vulnerabilities and to design precision therapeutic strategies that disrupt nutrient flow and regulatory networks existing between malignant and stromal compartments.
